# Atomic collisions in suprafluid helium-nanodroplets: timescales for metal-cluster formation derived from He-density functional theory

**DOI:** 10.1039/c5cp01110h

**Published:** 2015-03-27

**Authors:** Andreas W. Hauser, Alexander Volk, Philipp Thaler, Wolfgang E. Ernst

**Affiliations:** a Institute of Experimental Physics , Graz University of Technology , Petersgasse 16 , A-8010 Graz , Austria . Email: andreas.w.hauser@gmail.com ; Email: wolfgang.ernst@tugraz.at

## Abstract

Collision times for the coinage metal atoms Cu, Ag and Au in He-droplets are derived from helium density functional theory and molecular dynamics simulations. The strength of the attractive interaction between the metal atoms turns out to be less important than the mass of the propagating metal atoms. Even for small droplets consisting of a few thousand helium atoms, the collision times are shortest for Cu, followed by Ag and Au, despite the higher binding energy of Au_2_ compared to Cu_2_.

## Abstract 2

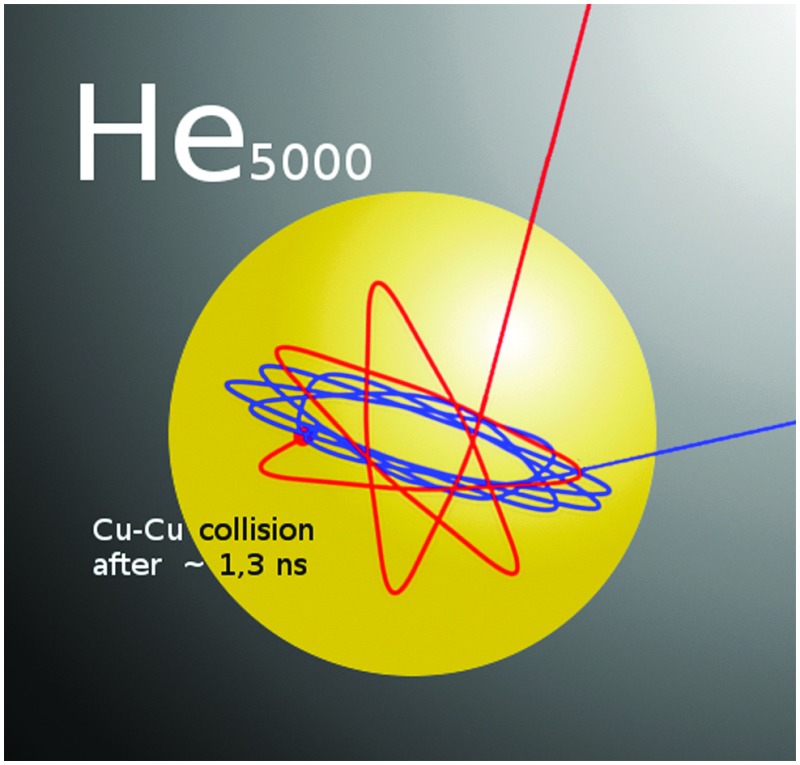
Two copper atoms, collected in a pickup-process by a He droplet consisting of 5000 atoms, move along rosetta-like, planar trajectories until the inter-particle attraction makes them collide.

## 1 Introduction

Metal clusters containing a few hundred or thousand atoms have experienced a tremendous interest in recent years due to their numerous potential applications in catalysis,^1^ optics^2^ and magnetics^3^ industries. However, bridging between isolated, single atoms and the bulk material, their properties are heavily dependent on particle size and structure,^4,5^ which leads to high demands on current synthesis techniques. In this context, superfluid helium droplets (He_*N*_) provide a novel, extraordinary tool for controlled particle growth, combining the advantage of a minimally interactive confinement at 0.37 K with versatile doping techniques that allow for a fine-tuned synthesis of pure or mixed metal clusters.^6–9^ First steps towards industrial applications such as the deposition of He_*N*_-grown clusters, cluster films or nano wires onto surfaces, were taken recently.^10–17^

Despite several theoretical studies of the interaction between a coinage metal dopant and the He environment,^18,19^ information on He-mediated cluster formation processes for coinage metals is yet very scarce. Of particular interest to the community is the knowledge about timescales for cluster formation, since it is crucial for a controlled growth of nanoparticles in helium droplets. Related studies of cluster formation in bulk helium describe a different growth mechanism, which is initiated by laser ablation of immersed metal targets and accompanied by the creation of vortices.^20–22^

As a first step towards this goal it will be necessary to investigate the motion of two dopants within a droplet of a given size. In this article, we apply He density functional theory to describe the initial mechanism of any cluster formation, namely the collision of two metal atoms. We pick the coinage metals Cu, Ag and Au (denoted as X throughout the article) for our study, since several experiments have been performed with these elements in our group recently.^10,23^ The necessary interaction potentials are taken from previous studies in the case of He–X or are generated by ourselves *via* high-level *ab initio* calculations in the case of the X–X potential curves. This manuscript is structured as follows. Section 2 is dedicated to technicalities of our approach. We discuss the diatomic potentials, the DFT approach and the molecular dynamics simulation. A correction for the X–X interaction potential is suggested, which takes the He-environment into consideration. In Section 3, we present results for the confinement potentials and use them together with the corrected intermetallic potentials for the simulation of atomic collision processes. A statistical analysis is given, including predictions for average collision times in case of Cu, Ag and Au capture. The results are compared to previous studies and experiments where possible.

## 2 Theory

Our computational approach can be divided into three steps, which will be discussed in separate sections: the *ab initio* calculation of the necessary diatomic potentials, the creation of density profiles and solvation energies for doped He droplets of various size, and the simulation of dopant movements within a droplet *via* molecular dynamics (MD).

### 2.1 He–X and X–X interactions

**Fig. 1:**
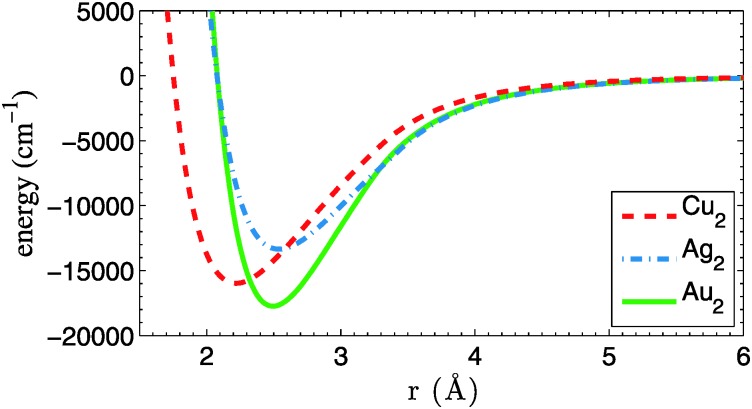
Potential energy curves for Cu_2_, Ag_2_ and Au_2_, calculated at the CCSD(T) level of theory. All curves are corrected for BSSE and have been fitted with an *r* ^–6^ dependence at long range.

A first ingredient are the pair potentials for He–Cu, He–Ag and He–Au, which are needed as input for a He–DFT code that generates relaxed He density distributions for larger droplets. Fortunately, the corresponding potential energy curves are available at high accuracy from ref. 24 and do not need to be calculated here. The corresponding binding energies are 6, 7 and 15 cm^–1^ for He–Cu, He–Ag and He–Au, respectively. Potential energy curves for the Cu_2_, Ag_2_ and Au_2_ metal dimers, on the other hand, are calculated by us at the coupled-cluster level of theory. They do not enter the DFT calculation, but are needed later for the MD simulation. A single-reference, partially spin-restricted open-shell variant of the coupled-cluster method with single and double excitations plus perturbative triples [RHF-RCCSD(T)] is employed,^25,26^ as it is implemented in the Molpro program package.^27^ The aug-cc-pVQZ-PP basis sets of Peterson *et al.* ^28^ are used together with their corresponding effective core potentials, which replace all but the outmost 19 electrons of each metal atom.^29^ All calculations are corrected for basis set superposition errors (BSSE) *via* the counterpoise method of Boys and Bernardi.^30^ The resulting dimer potential curves are plotted in Fig. 1. Accuracy at long interatomic distances is granted *via* 1/*r* ^6^ fits to the atomic asymptotes. Binding energies *D*_e_, equilibrium bond lengths *r*_e_ and harmonic frequencies *ω* are summarized in Table 1 and compared to experimental data. The frequencies are derived from the first few vibrational levels of the *ab initio* potential curves. A symmetric three-point finite difference method has been used to solve the one-dimensional Schrödinger equation for the nuclear motion. All parameters are in good agreement with previous theoretical^31^ and experimental studies.^32^

**Table 1:** Parameters of the X–X potential energy curves. Results of the present work (pw) are compared to experimental data taken from ref. 32

PES	*r*_e_ (Å)	*D*_e_ (cm^–1^)	*ω* (cm^–1^)	Ref.
Cu–Cu	2.22	15 974	264	pw
Cu–Cu	2.22	16 534	265	Exp.
Ag–Ag	2.54	13 345	188	pw
Ag–Ag	2.53	13 389	192	Exp.
Au–Au	2.50	17 737	182	pw
Au–Au	2.50	18 551	191	Exp.

### 2.2 Dopant solvation in He_*N*_

#### 2.2.1 Single atom solvation

Free energies and density distributions for doped droplets of varying size are obtained from a He density functional approach, which accounts for a one-sided interaction between dopant and He droplet. We apply the Orsay-Trento-density functional^33^ to map the He density onto the energy, using the FORTRAN code of F. Dalfovo with modifications of Lehmann and Schmied.^34^ *F*[*ρ*], the free energy of a doped He droplet, is minimized with respect to a fixed dopant position by evaluation of the He density distribution on a cylinder symmetric grid of cylinder coordinates *z* × *r*, spanning over 601 × 300 points with a spacing of 0.238 Å. It can be written as a functional of the He density *ρ*, according to the formula:^35^

1 *F*[*ρ*] = *E*[*ρ*] + *U*_ext_[*ρ*] – *μN*[*ρ*],

with *E*[*ρ*] denoting the Orsay-Trento functional and *U*_ext_ as the external potential. The latter introduces the interaction between He and the dopant, and is generated by a summation over energy contributions from the corresponding He–X pair potential of the previous subsection at the various distances between dopant and the He density distribution on the given grid. The third term in eqn (1) accounts for the conservation of the total helium amount, and consists of *N*[*ρ*], the number of He atoms, multiplied by its corresponding Lagrange parameter, the chemical potential *μ*.

#### 2.2.2 Solvation effects on X–X interactions

We further use the DFT approach of above to study effects of the superfluid helium environment on interatomic interactions by immersing two metal atoms into the droplet, followed by a re-evaluation of their dimer potential energy curve as a function of distance. Note, however, that the DFT code does not account for any direct interaction between two metal atoms. Therefore, the only energy dependence that can be derived from this computational experiment is a description of how the He_*N*_ droplet energy is affected by the helium density disturbance (*i.e.* the two ‘density holes’) caused by the immersion of X–X. If we remain with the assumption of negligible three-body-interactions in the given study (which is the fundamental assumption behind the well-established pair summation technique of He–X interactions anyway), this allows us to correct the X–X potential curves in a simple manner: we put two dopants into the middle of a He_*N*_ droplet and calculate the energy of the doped droplet as a function of the interatomic distance between the two dopants with the asymptotic value set to zero. The ab initio-derived X–X curves are then corrected by this extra energy contribution that stems only from the He density rearrangement caused by the X–X bond length variation:2
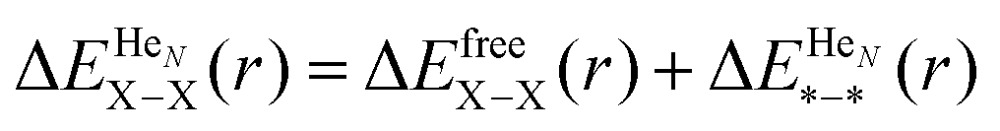
The stars in the last term represent the density holes caused by the He–X interactions. Note that for droplets of 1000 He atoms and larger the potential energy of a single dopant X with respect to its absolute position within the droplet is almost constant (as will be shown in the Results section) except for near-surface positions, and can therefore be neglected here.

### 2.3 MD simulation of dopant motions

In the final step we combine the He–X and X–X interactions of the previous sections to calculate trajectories for the metal atoms immersed in He_*N*_ by solving Newton's equations of motion in a classic picture. We apply the Velocity-Verlet algorithm with a fixed time step of 0.1 picoseconds. The He–X interactions create a spherically symmetric confinement potential which keeps the dopants within the droplet. This potential has its minimum at the center of the droplet. Additionally, the metal atoms themselves interact as described by the corrected X–X potential. The actual motion of the dopants through liquid helium is accounted for in a simple manner: their velocity can not overcome the Landau limit *v*_*λ*_ ≈ 56 m s^–1^ at any time during the simulation. Recently, it has been shown that such a critical velocity is existent even for droplets consisting of only a thousand He atoms.^36^ Such a limitation of the velocity during the simulation addresses the fact that friction, appearing for velocities *v* > *v*_*λ*_, leads to the immediate dissipation of the excess kinetic energy by the creation of roton pairs. The generation of these quasiparticles, which correspond to local excitations of the He density, reduces the dopant velocity until it drops below the Landau level.^37,38^

Initial positions are randomly distributed within the droplet, and the particle velocities are chosen from a uniform distribution in the interval from 0 to *v*_*λ*_. This grants an unbiased sampling, but also allows for some particles to leave the droplet, which we dismiss from the statistics since they can not be considered ‘captured’. A particle is considered as lost if it shows a trajectory that leads out of the He droplet environment. We define the latter as the volume inside a sphere of radius *R* + *r*_eq_, with *R* chosen as the distance where the He density drops below 1/*e* of its bulk value, and *r*_eq_ = 4.5 Å as an average value for the He–X equilibrium distances. For the larger droplets this restriction leads to a sampling loss of less than one percent.

## 3 Results and discussion

### 3.1 Interaction potentials and solvation effects

**Fig. 2:**
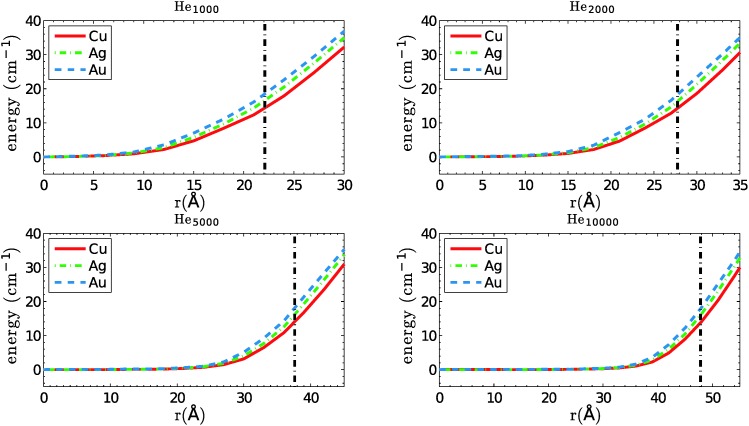
Confinement potentials for Cu, Ag and Au in helium droplets consisting of 1000, 2000, 5000 and 10 000 atoms. The estimated droplet radius is indicated by a straight, dash-dotted line.

We start with a comparison of confinement potentials obtained for Cu, Ag and Au, as a function of the distance to the center of mass of the total helium density. They are given in Fig. 2. If we assume a maximum velocity of *v*_*λ*_ for the dopant atoms, their kinetic energies are always below 8, 14 and 26 cm^–1^ for Cu, Ag and Au, respectively. Note that the reflection points for silver atoms with velocities near *v*_*λ*_ lie very close at the droplet surface, and for gold atoms they are even outside of the valid sampling range defined above. These heavy atoms are able to leave the region of high He density behind and move beyond the droplet radius even if their trajectory goes through the droplet center. However, from the same table it can be seen that even in these cases the dopant energies are well below the corresponding solvation energies, meaning that the atoms are still not able to fully detach from the helium droplet. They are dragged back *via* long-ranging van der Waals interactions, and get fully immersed into the droplet again. These interesting cases are probably worth a study on their own, but will be skipped here for two reasons. One argument is that such a surface-interacting or ‘diving’ trajectory might be poorly described with classical methods, as is necessitates a dynamic description of the He density distortion created on the droplet surface. The other one is that droplets of a size where the sampling loss is not marginal are barely able to capture such a heavy atom in the first place. Recently, a closely related type of translational dynamics was investigated for photoexcited Ag atoms on small He droplets (*N* = 1000) *via* time-dependent density functional theory.^39^

Solvation energies, defined as the energy difference between the doped and the undoped droplet *via* the equation

3 *S*(*M*) = *E*(He_*N*_ + *M*) – *E*(He),

are listed in Table 2. Absolute values of the solvation energies increase with the size of the droplet. As expected from the pair potential depths, He_*N*_–Au gives the largest absolute value, followed by Ag and Cu.

**Table 2:** Solvation energies (cm^–1^) of Cu, Ag and Au in helium droplets of various size. Droplet radii are given in Å

Dopant	He_1000_	He_2000_	He_5000_	He_10000_
Cu	63.2	64.0	64.3	64.6
Ag	85.2	85.9	86.3	86.5
Au	175.6	175.8	175.6	175.5
He_*N*_ radius^*a*^	22.11	27.76	37.61	47.83

^*a*^Radial distance from center of He_*N*_ mass where the density drops below 1/*e* of the bulk value *ρ* = 0.02185 Å^–3^.

**Fig. 3:**
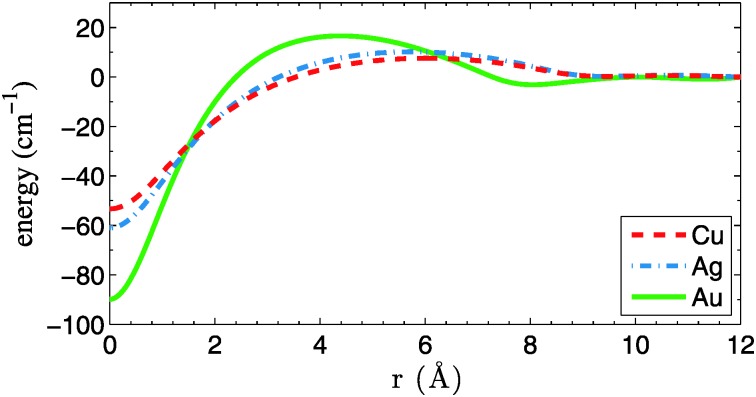
Correction energies for the PES of Cu_2_, Ag_2_ and Au_2_ in He_5000_, as a function of the dimer bond length.

**Fig. 4:**
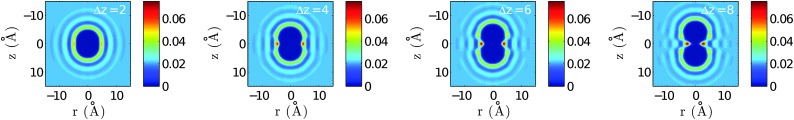
Helium density distribution of an Ag_2_ doped droplet for various interatomic Ag–Ag distances (denoted as Δ*z*). The local distortion causes a series of damped oscillations in the nearby He density. Note also the maxima of the He density in the mirror plane of the molecule, perpendicular to the internuclear axis (*i.e.*, the *z*-axis). Densities are given in units of Å^–3^.

In the next step we discuss corrections to the X–X potentials. The correction functions 
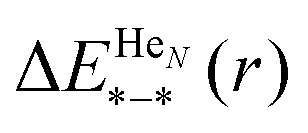
 are plotted in Fig. 3 for doped droplets consisting of 5000 He atoms. The asymptotic value is set to zero for all curves. Energies were calculated at steps of 0.5 Å. All curves show minima for overlapping atoms, which seems counterintuitive at first sight, since such a geometry represents a minimum of the contact surface between the dopant atoms and the surrounding He density, while two dopants, embedded in separate bubbles, would correspond to a maximum of the contact surface area. From this finding we derive that the minimization of local distortions in the helium distribution overcompensates the loss of contact surface to the surrounding helium. Therefore, the X–X potential curves experience a slight contraction when corrected for the presence of helium. However, the corrections are very small compared to the overall dimer binding energies, and the geometry effects are completely negligible. We note that similar He density effects play a much bigger role in cases of weak diatomic interactions, *e.g.* for Rb–Xe in He_*N*_.^40^ Interestingly, the curves show a maximum at finite distances (6.0, 5.7, and 4.4 Å for Cu, Ag and Au) and a slight oscillation towards the asymptote, which we explain by the spherical density fluctuations around each dopant. These fluctuations are illustrated in Fig. 4, which contains a series of density plots for Ag–Ag distances from 2 to 8 Å. An interesting feature besides the known oscillations of the He density in the nearby region is the formation of a donut-shaped ring of higher He density, which gets more pronounced for larger internuclear distances and appears as two separate maxima in the two-dimensional density cuts. Similar effects were reported recently for chains of atoms pinned to a vortex in superfluid He.^41^ We note that this phenomenon of increased density at small internuclear distances and the related energy penalty could have an effect on collision probabilities of weakly interacting particles. If the correction energy as shown in Fig. 3 is not overcompensated by the attractive interaction, a barrier will remain, and collision times obtained from the classical picture might have to be corrected for the effect of quantum tunneling.

### 3.2 Dopant trajectories and average collision times

**Fig. 5:**
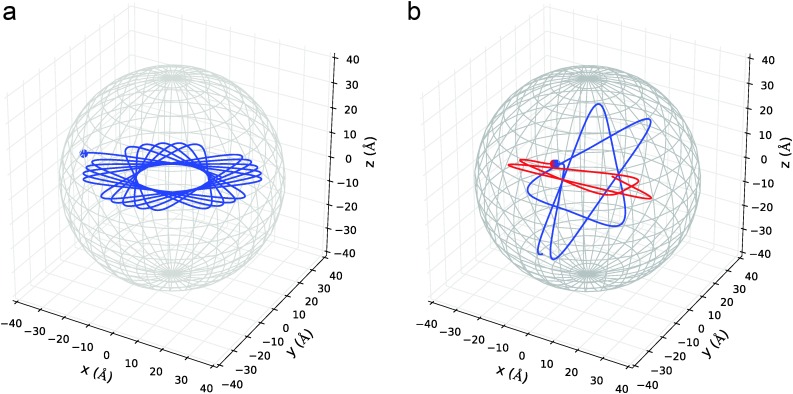
Typical trajectories for a single (left) or for two atoms (right), given the example of Cu dopants in He_5000_. Note the conservation of the angular momentum in the case of a single atom deposition, which leads to a rosetta-like, planar trajectory. In the right picture, where the two atoms attract each other, only the total angular momentum is conserved. Both particles move independently in their planes until the internuclear distance is accidentally small enough for the attractive interaction to force them on a collision course.

In this section we present the results of the MD simulations. Example trajectories for Cu in He_5000_ are given in Fig. 5, where the cases of single and double deposition are depicted. In the case of a single atom deposition, the angular momentum of the particle is conserved, and its trajectory is therefore always planar. This symmetry is removed as soon as a second atom is introduced to the system, and their trajectories are forced out of plane due to the interatomic Cu–Cu interaction.

10^4^ collisions have been simulated for each metal dopant and each droplet size. The results for the average collision times are summarized in Table 3. The evaluation of Ag and Au in He_1000_ has been skipped due to the higher loss of particles during the simulation and a strong dependence of the collision times on our definition of the valid trajectory range. Collision times rank from about 0.3 to 4 nanoseconds in this size regime. We find that even for He_2000_ droplets, with radii of less than 28 Å, the collision times are not ranked according to the depth of the X–X interaction potential. The plausible assumption of higher binding energies leading to shorter collision times holds only for particles of similar mass. For the dopants chosen here, with mass ratios of roughly 2 : 3 : 6, the time ranking correlates with the particle mass for all droplet sizes. Copper collisions happen fastest, followed by silver and gold. The more energetic, heavier atoms propagate on average through a larger volume of the droplet, since they are reflected further outside by the confinement potential. As can be seen from Fig. 2, the kinetic energy difference between Cu (8 cm^–1^) and Au (26 cm^–1^) translates into a difference of about 10 Å for their point of reflection.

**Table 3:** Collision times (picoseconds) for pairs of Cu, Ag and Au atoms in helium droplets of various size

Dopant	He_1000_	He_2000_	He_5000_	He_10000_
Cu	316	515	1274	2620
Ag^*a*^	—	928	1708	2843
Au^*a*^	—	1374	2266	3990

^*a*^The average collision times for gold and silver in He_1000_ have been skipped since they are strongly biased by the cutoff definition (see text).

**Fig. 6:**
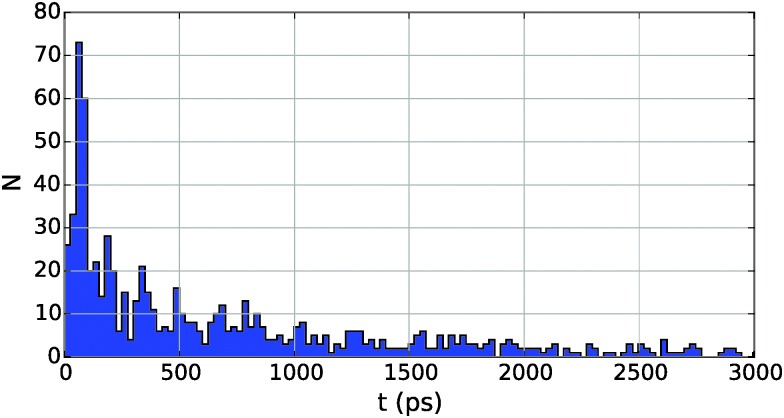
Collision times for Cu in He_5000_. The long tail of the probability density has been cut to emphasize details. Note the periodicity of about 130 picoseconds between peaks.

Interestingly, the probability density for collision events over time shows a slight periodicity, as can be seen from the example histogram given in Fig. 6 for Cu_2_ in He_5000_. The peak-to-peak distance, in this case about 130 picoseconds, is the approximate time needed for a particle at *v*_*λ*_ to traverse the droplet. This relationship also holds for larger and smaller droplets, leading to longer and shorter intervals, respectively.

### 3.3 Extrapolations to larger droplets

**Fig. 7:**
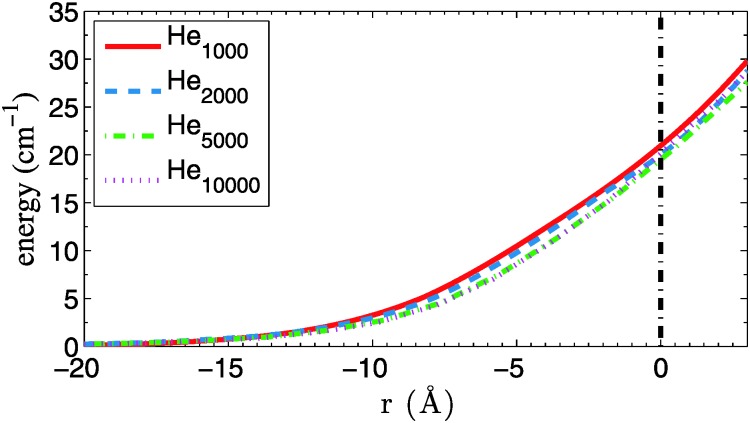
Confinement potentials for Cu in He droplets of various size. The zero position is set to the point where the He density drops below 1/*e* of the bulk value (vertical, dashed line). On this *x*-axis, the droplet centers lie at –*r*, with *r* taken from Table 2.

We extend our MD analysis to larger He droplets with radii up to 100 nm, which play a much bigger role in the ongoing experiments on metal cluster formation for the following reason: from our DFT simulations we obtain a chemical potential of about 4 cm^–1^ for He, which means that upon formation of our metal dimers about 3000 to 4000 He atoms have to be dissipated after the atomic collision. Therefore, cluster formation processes can only take place in droplets with larger radii. DFT simulations are currently too expensive in this size regime, but we can take advantage of the fact that the curvatures of the confinement potentials for the same metal but different droplet sizes are almost identical in the relevant energy range. This is illustrated in Fig. 7, where the confinement potentials for Cu in He_*N*_ are compared to each other. Following the definition of the droplet radius as given in Table 2, and setting this value to zero for each droplet, one finds almost overlapping confinement curves, as far as the curvature near the surface is concerned. From this we conclude that confinement potentials for larger droplets (*N* > 10 000) can be easily obtained by simple shifts of the He_10000_ potential to larger distances *r*. We repeat our simulations for larger droplets with radii of 100, 200, 500 and 1000 Å, respectively.

In our MD simulation, the most time consuming step is the evaluation of the potential energy gradient at each timestep. In large droplets, this evaluation is not necessary most of the time, since the particles move on straight lines. This is taken into consideration in the code by reducing the evaluation to cases where the inter-particle distance drops below 30 Å or when they are closer than 15 Å to the droplet surface. However, a much more significant reduction of computational costs can be achieved by a complete avoidance of gradient evaluations for the confinement potential. Benchmark calculations for He droplets with a radius of 200 Å show that at this size the confinement potential can be replaced by a simple hard wall reflection, since the time spent in the areas with potential energy *U*_pot_ > 0 is small compared to the free motion (*U*_pot_ = 0) through the droplet. For better agreement with the more accurate model the actual position of the hard wall is chosen with respect to the atom type as follows: assuming a shifted soft potential as described in the text, we determine the point of reflection for atoms with velocity *v*_*λ*_/2, and place the hard wall at this position. For droplets with a radius of 1000 Å, for example, the reflective wall is placed at 991, 993 and 995 Å for Cu, Ag and Au, respectively. This way, the simplified model also accounts for the larger accessible volume of heavier atoms. The difference in collision time predictions for droplets with a radius of 200 Å compared to results obtained with the soft potential is less than 5%. We therefore apply this simplified approach to the largest droplets with radii of 500 and 1000 Å. Our results for the average collision times in this size regime are summarized in Table 4. For the largest, and experimentally most relevant droplets with a radius of 1000 Å, we obtain collision times in the range of 0.011–0.014 ms.

**Table 4:** Collision times (nanoseconds) for pairs of Cu, Ag and Au atoms in larger helium droplets, sorted by their radius

Dopant	100 Å	200 Å	500 Å	1000 Å
Cu	16	114	1352	11 031
Ag	18	121	1418	11 401
Au	24	163	1790	14 452

### 3.4 Comparison to other models

To our knowledge, this is the first evaluation of collision times *t*_coll_ for coinage metal atoms in He_*N*_ accounting for interactions between two dopants as well as between dopants and the helium droplet itself. However, knowledge about timescales of cluster formation are the key to a better understanding of complex growth processes observed in recent experiments, such as multicenter growth or the creation of nanowires.^10–12,15–17^ Therefore, several model descriptions have been used in the past, which can be compared to our calculations.

**Fig. 8:**
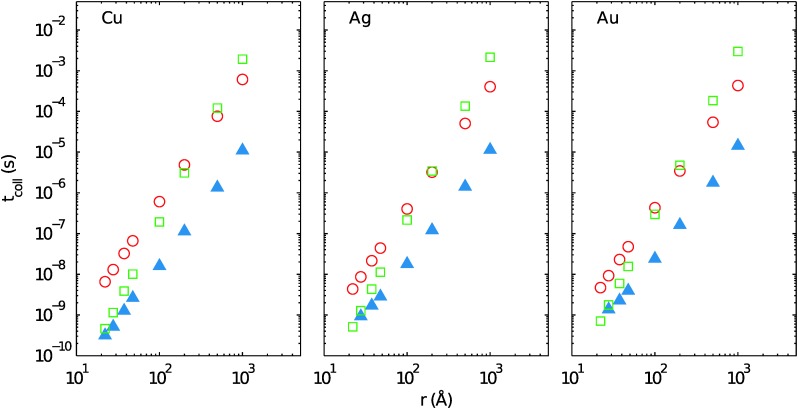
Collision times of coinage metals in He_*N*_ as obtained with different models, either by dividing the total He_*N*_ volume by the product of particle velocity and cross section as proposed in ref. 43 (open circles), by assuming pure van der Waals attraction as proposed in ref. 42 (open squares), and from the calculations in this work (full triangles). See text for further discussion.

In ref. 42, multiple dopant pickup and successive coagulation of gas atoms and molecules were investigated by mass spectroscopy and Monte Carlo simulations. Assuming that two successively collected dopants come to rest at random positions within a droplet, *t*_coll_ is calculated as being only dependent on the inter-particle van der Waals forces, thereby neglecting remaining kinetic energies and the influence of the He environment. Another approach to estimate the onset of multicenter aggregation is given in ref. 43, where the coagulation time for two particles is approximated as the time it takes to sweep the collision cross section of the particles through the total volume of the droplet at constant velocity. Both aforementioned formalisms were applied to droplet sizes and dopants considered in this work. For the first model, the corresponding *C*_6_ coefficients of the –*C*_6_/*r* ^6^ van der Waals potentials are derived from the dimer curves given in Fig. 1. We average over several simulation runs with initial distances randomly chosen within the droplet diameter. For the second model, we assume an average velocity of *v*_*λ*_/2. The results are comparatively depicted in Fig. 8.

One finds that the values for *t*_coll_ in the present work are generally lower and the agreement with the simpler models seems to get worse with increasing droplet size. Considering only van der Waals interaction, this seems to be intuitive as the attractive potential between the dopants is proportional to *r* ^–6^. Still, there is a good agreement between the van der Waals-only model and our calculation for small He_*N*_ sizes. Collision times obtained from the static volume model are more than one order of magnitude larger and show practically no dependence on the atom type. In the latter feature the simple model agrees with our calculations for large droplets, where the X–X interactions become less relevant. The slopes of the volume model and our calculation are almost parallel. Obviously, one reason for the overestimation of collision times lies in the complete neglect of interatomic attractive interactions. However, this argument does not explain the still significant discrepancy for larger droplets. The additional, and apparently more relevant deviation that remains in the case of larger volumes stems from the fact that the trajectory of an essentially unaffected, confined particle is planar due to the conservation of the angular momentum. Therefore, the assumption of the whole volume being accessible must lead to an overestimation of collision times.

## 4 Conclusion

We simulated the motion of Cu, Ag and Au atoms in droplets of superfluid helium *via* a combination of He-density functional theory and classical molecular dynamics. The necessary two-particle interaction potentials were either taken from literature or derived from quantum chemistry calculations at the CCSD(T) level of theory. The metal dimer potentials were corrected for energy penalties which arise due to local distortions of the helium density. However, these corrections turn out to be fully negligible (less than 100 cm^–1^) for the strongly bound metal dimers and do not affect their equilibrium geometries, but could become relevant for weakly bound species such as Mg_2_. The confinement potentials were calculated with our DFT code for He droplets consisting of up to 10 000 atoms. We found that the shapes of these potentials are minimally affected by the droplet size, which allowed the simulation of larger droplets with radii of up to 100 nm by simple shifts of the curves.

In a series of molecular dynamics simulations for helium droplets with radii from 23 to 1000 Å we calculated the trajectories of two metal atoms in a symmetric confinement *via* a Velocity-Verlet integration upon collision. A statistical analysis of collision times for the various helium droplet sizes after the pickup of a second metal atom shows that the strength of the metal–metal interaction is overcompensated by particle mass effects even in small droplets consisting of a few thousand helium droplets (radii below 30 Å). On average, Cu collisions are slightly faster than Ag and Au collisions.

Our findings should be useful to experimentalists for basic estimates of more complex cluster growth scenarios in helium droplets of various size, where collision events, pickup processes and other phenomena such as vortex-induced nanowire formation have to be taken into consideration. An extension of our theoretical studies towards the simulation of actual growth mechanisms in combination with experiments on coinage-metal-doped He droplets is in preparation.
